# Identification of Modules Related to Programmed Cell Death in CHD Based on EHEN

**DOI:** 10.1155/2014/475379

**Published:** 2014-07-15

**Authors:** Xu Jia, Wan Li, Zhengqiang Miao, Chenchen Feng, Zhe Liu, Yuehan He, Junjie Lv, Youwen Du, Min Hou, Weiming He, Danbin Li, Lina Chen

**Affiliations:** ^1^College of Bioinformatics Science and Technology, Harbin Medical University, Harbin, Heilongjiang 150000, China; ^2^Institute of Opto-Electronics, Harbin Institute of Technology, Harbin, Heilongjiang 150000, China; ^3^Surgery, The First Affiliated Hospital of Harbin Medical University, Harbin, Heilongjiang 150000, China

## Abstract

The formation and death of macrophages and foam cells are one of the major factors that cause coronary heart disease (CHD). In our study, based on the Edinburgh Human Metabolic Network (EHMN) metabolic network, we built an enzyme network which was constructed by enzymes (nodes) and reactions (edges) called the Edinburgh Human Enzyme Network (EHEN). By integrating the subcellular location information for the reactions and refining the protein-reaction relationships based on the location information, we proposed a computational approach to select modules related to programmed cell death. The identified module was in the EHEN-mitochondria (EHEN-M) and was confirmed to be related to programmed cell death, CHD pathogenesis, and lipid metabolism in the literature. We expected this method could analyze CHD better and more comprehensively from the point of programmed cell death in subnetworks.

## 1. Introduction

Coronary heart disease (CHD) is the narrowing or blockage of the coronary arteries, usually caused by atherosclerosis. Atherosclerosis is the buildup of plaques on the inner walls of the arteries, which can restrict blood flow to the heart muscle by physically clogging the artery or by causing abnormal artery tone and function [1]. In general, apoptosis occurring in atherosclerotic lesions has been suggested to be involved in the evolution and the structural stability of the plaques [2]. Because late apoptotic cells can launch proatherogenic inflammatory responses, adequate engulfment of apoptotic cells (efferocytosis) by macrophages is important to withstand the atherosclerosis progression [3]. Macrophages represent more than 40% of dead cells in the atherosclerotic lesions [4]. Enhancing intracellular lipid content can enlarge foam cells formation [5]. Formation of lipid-laden foam cells from macrophages and, to a less extent, from smooth muscle cells represents a landmark for atherosclerosis [6]. Some studies have documented that deregulation of apoptosis, a form of genetically programmed cell death, occurs in atherosclerotic lesions [7, 8].

Programmed cell death (PCD) is proposed to be death of a cell in pathological format, mediated by an intracellular program [9]. PCD referred to apoptosis, autophagy, and programmed necrosis. These three forms of PCD may jointly decide the fate of cells; apoptosis and programmed necrosis invariably contribute to cell death, whereas autophagy can play either prosurvival or prodeath roles [10]. Imbalance between cell survival and death may contribute to dramatic alterations in cellularity of the arterial wall with atherosclerosis. Carotid Atherosclerosis Progression Trial Investigating Vascular Cholesterol Acyltransferase (ACAT) Inhibition Treatment Effects was developed to assist in the prevention of cardiovascular disease [11]. Cyclooxygenase-2 (COX-2) in control of cell proliferation, apoptosis, played a significant role in the development of atherosclerosis [12, 13]. Matrix metalloproteinase produced by macrophages played an important role in modulating plaque stability and apoptosis of cell [14, 15]. Macrophages play a crucial role at all stages of CHD, including regulation of foam cell formation, the inflammatory response, and the stability of atherosclerotic plaques. The effect of enzyme in macrophages and foam cells is of great value in the study of CHD from the perspective of programmed cell death.

Traditional experimental research was through single gene or single compound to analyze CHD, and it could not provide the analysis at system level. Along with the rapid development of technology, the study about biological networks is increasingly providing valuable information on biological systems [16–18]. Metabolic network is an important typical biochemical network which consists of enzymes and chemical compounds [19, 20]. Due to localization of metabolic enzymes, many metabolic processes involve coordinated interactions between different organelles, and one metabolic step may be dependent upon the successful completion of the previous step [21, 22]. In other words, chemical compounds can be considered as edges mediating between enzymes. A better understanding of human metabolism and its relationship with diseases is an important task in human systems biology studies. In metabolic reactions, there is a cascade relationship between enzymes. The relationship network between enzymes was constructed to select risk enzymes related disease and reveal the pathogenesis. Considering from this aspect, enzyme marker will be searched and be used for disease detection in the near future.

Therefore, in our paper, we presented a high-quality human metabolic enzyme network, Edinburgh Human Enzyme Network (EHEN), manually reconstructed based on Edinburgh Human Metabolic Network (EHMN) [23], and divide it into different subnetworks, by integrating genome annotation and location information from different databases and metabolic reaction information from the literature. We presented a strategy to select reporter enzyme considering the change of foam cells and macrophages between normal state and disease state with network characteristic. Through the method of selecting modules based reporter enzymes and the functional enrichment analysis, the module related to programmed cell death was analyzed further. We expected this method could analyze CHD better and more comprehensively from the point of programmed cell death in subnetworks.

## 2. Materials and Methods

### 2.1. Gene Expression Data Source and Data Preprocessing

We used monocyte-derived macrophages from peripheral blood cultured in the absence or presence of oxidized LDL, baseline macrophages or foam cells. The macrophages were obtained from 15 subjects with subclinical atherosclerosis and a family history of CHD. Macrophages from 15 age and gender matched subjects with no atherosclerosis and no family history of CHD were used as control. We downloaded this expression data with the accession number of GSE9874 [24] from NCBI-GEO database (http://www.ncbi.nlm.nih.gov/geo/).

### 2.2. Construction of Enzyme Network and Enzyme Subnetwork Based on EHMN

**Figure 1:**
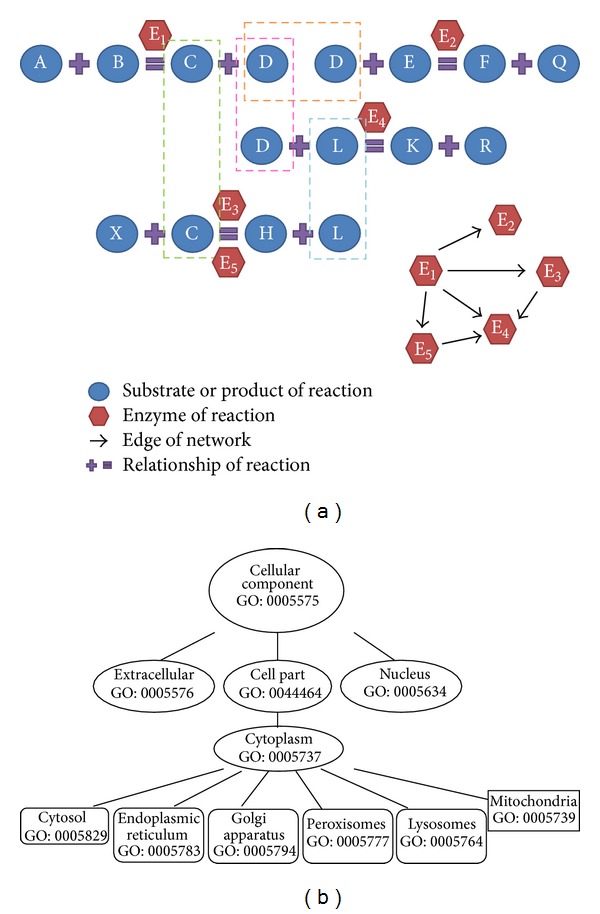
Schematic diagram of enzyme network construction and protein location method. (a) Substrates and products of reactions are shown as blue circles, enzymes of reactions are shown as red hexagons, relationships of reaction are shown as purple mathematical symbols, edges of network are shown as black arrows, and the arrow pointing means the order of reactions. (b) The Gene ontology terms used for protein locations. The top locations are circled. Proteins are matched to GO terms and then backtracked to the selected locations through the hierarchical structure.

The Edinburgh Human Metabolic Network (EHMN) was reconstructed by integrating genome annotation information from different databases and metabolic reaction information from the literature [23]. EHMN contains nearly 3000 metabolic reactions, which were reorganized into 70 human-specific pathways according to their functional relationships [25, 26]. Based on EHMN metabolic network, we built an enzyme network which was constructed by enzyme (nodes) and reactions (edges). According to reaction relationships between substrates and products (i.e., the product of a reaction is just right the substrate of the next reaction), corresponding enzymes of reactions were connected to a network, as shown in Figure 1(a). Because the enzyme network construction is based on EHMN, the Edinburgh Human Metabolic Network, we called it the Edinburgh Human Enzyme Network (EHEN).

The enzyme protein location information in this work is extracted mainly from gene ontology (GO) [27]. Files containing GO association in human and the hierarchically organized GO terms (“OBO” file) can be easily downloaded from the GO website. This file is maintained by the GOA group at EBI which aims to provide high-quality GO annotations to proteins in the UniProtKB, as demonstrated in Figure 1(b). The proteins with uncertain locations or other locations not included in the known specific locations were classified to an “uncertain” location, and they will be finally screened out from our study.

### 2.3. Selection of Modules Related to Programmed Cell Death Based on Reporter Enzymes

We proposed a computational approach to select modules related to programmed cell death. All the disease and matched control samples were normalized simultaneously using the robust multiarray average (RMA) method [28], implemented in R/Bioconductor project. We calculated and obtained risk enzymes from the above expression profile of foam cells and macrophages, separately. Next, the common enzymes of two cells were sought as reporter enzymes. Further, the network functional modules related to programmed cell death based on reporter enzymes were selected. Details of our selection progress are as follows.

1. *P* values of genes, *P*_value_*g*_, indicating the significance of differential expression, were converted to standard *Z*-scores *Z*_value_*g*_ with a mean of 0 and a variance of 1 by using the inverse normal cumulative distribution function (CDF) (*θ* ^−1^):
  (1) $$\begin{array}{c} Z\_\text{valu}{\text{e}}_{g}=\text{cd}{\text{f}}^{-1}\left(1-P\_\text{valu}{\text{e}}_{g}\right). \end{array}$$
2. *Z*-scores of enzymes calculated as median of *Z*-scores of the *k* genes were as follows:
  (2) $$\begin{array}{c} {Z\_\text{value}}_{\text{Enzyme}}=\frac{1}{k}\overset{n}{\underset{i=1}{\sum }}\left\{Z\_\text{valu}{\text{e}}_{g}\right\}. \end{array}$$
3. *Z*_value_Enzyme_ scores were then corrected for the background distribution by subtracting the mean (*μ*_*k*_) and dividing by the standard deviation (*σ*_*k*_) of the aggregated *Z*-scores derived by sampling 10000 sets of *k* enzymes from the EHEN:
  (3) $$\begin{array}{c} Z_{\text{Enzyme}}^{\text{corrected}}=\frac{Z_{\text{Enzyme}}-\mu_{k}}{\sigma_{k}}. \end{array}$$
4. Corrected *Z*-scores were then transformed to *P* values by using CDF:
  (4) $$\begin{array}{c} P\_\text{valu}{\text{e}}_{\text{Enzyme}}=1-\text{cdf}\left(Z_{\text{Enzyme}}^{\text{corrected}}\right). \end{array}$$
5. Reporter enzymes of the EHEN were screened with *P* values under a significant threshold of 0.05.
6. The network functional modules based reporter enzymes were found by MCODE [29] (with the parameters of degree cutoff ≥2 and *K*-core ≥2).
7. Gene ontology (GO) enrichment analysis was performed for each module from 6; select the modules related to programmed cell death.
8. To repeat the above method in the 6 subnetworks, we got the reporter enzymes related network functional modules based on different subnetworks. Finally, we got reporter enzymes related network functional modules in the EHEN and six subnetworks.

### 2.4. Functional Programmed Cell Death-Module Analysis of the EHEN and Subnetworks

In order to analyze the relationship between cell death and CHD, we select the functional modules related to programmed cell death for further research. GO functional enrichment analysis was applied for these modules using the Functional Annotation Tool in DAVID Bioinformatics Resources 6.7 (http://david.abcc.ncifcrf.gov/). FDR less than 0.05 was considered as significant.

## 3. Result

### 3.1. Enzyme Network and Subnetwork from the EHMN

Using the method described above in the network and subnetwork construction, all the human proteins coming from GO were classified into the chosen locations. By the described method, our constructed EHEN was separated into 6 subnetworks according to its localization information in our study, including cytoplasm (EHEN-C), mitochondria (EHEN-M), Golgi apparatus (EHEN-G), extracellular (EHEN-E), nucleus (EHEN-N), and endoplasmic reticulum (EHEN-R).

The input for network topological analysis to the calculation is the list of relationships of enzymes. Observing the degree distribution, characteristic path length, the connectivity, network diameter, the average and the maximum of the shortest path lengths, and clustering coefficient, the EHEN and subnetworks were found as scale-free following nearly a power law model (*f*(*x*) = *a*∗*x* ^*b*^,  *a* = 29.9,  and  *b* = −0.46) and had small-world properties with scale-free topology, which was a general characteristic of complex biological networks (more topological characteristics of networks are in the Supplementary Material Table S1 and Figure S1 (see Supplementary Material available online at http://dx.doi.org/10.1155/2014/475379)).

### 3.2. The Modules Based Reporter Enzymes in EHEN and Subnetworks

From the reporter enzyme selection algorithms in the method part, we got reporter enzymes of overall and subnetwork, respectively. The numbers of reporter enzymes varied in different networks; the EHEN-C had the largest number of reporter enzymes, while the EHEN-R had only 2 selected reporter enzymes; the overall EHEN network stayed in the middle position (7 reporter enzymes), as listed in the Supplementary Material (Table S2: the reporter enzymes of overall and subnetwork). The degree and the clustering coefficient of the reporter enzymes had significant difference (*P* < 0.05) in their respective networks. It was shown that the reporter enzymes had smaller degree and clustering coefficient in the EHEN, while they had bigger degree and clustering coefficient in subnetworks (more topological characteristics of reporter enzymes are in the Supplementary Material Table S3). It implied that the reporter enzymes selected from subnetworks played a more important part in the disease emergence and tended to form network modules.

**Figure 2:**
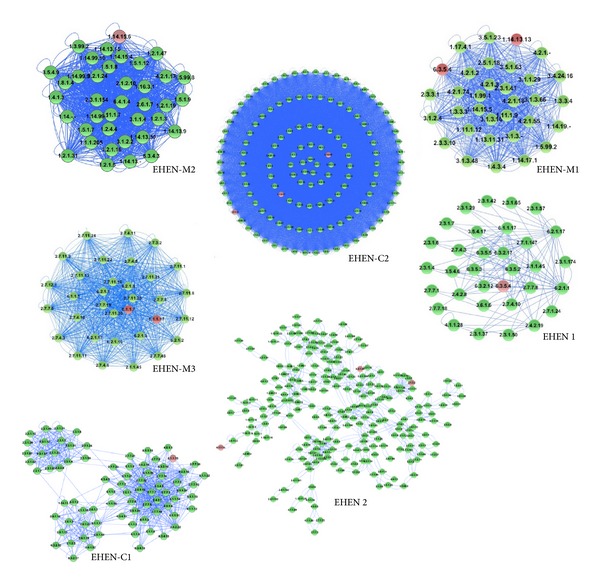
Seven modules based on reporter enzymes.

With the help of Cytoscape and its plug-ins, we selected out modules based reporter enzymes. From module division and annotation result of the EHEN and subnetworks by using Mcode, we gained functional modules of each network. Gene ontology (GO) enrichment analysis was performed for seven modules using DAVID, including 2 modules in the EHEN, 2 modules in the EHEN-C, and 3 modules in the EHEN-M (see Figure 2, and more information of modules in the Supplementary Material Table S4).

### 3.3. Literature Retrieval and Functional Annotation of the Reporter Enzyme and Related Modules

CHD was caused by the plaques buildup of lipid, which was created by macrophages forming foam cells. We defined the classification principles based on CHD-related functions-CHD pathogenesis, lipid metabolism, and programmed cell death (Table 1). Most of the modules could be enriched to the basic metabolic function class, the related CHD pathogenesis, and the related lipid function class. For instance, asparagine synthase was a reporter enzyme in both the EHEN and the EHEN-M and significantly inhibited the proliferation of cells [30]. In the module EHEN-M2, another reporter enzyme ec: 1.14.15.6, cholesterol monooxygenase (CYP11A1), also known as cytochrome P-450scc was the most important family of enzymes in microsomal mixed function oxidase, widely distributed in vivo, and it was detected human cardiac expression of the mRNAs for many of the enzymes involved in the formation of adrenal corticosteroids, supporting the possibility of local production of corticosteroids and a physiological role for these hormones in cardiac function [31]. In the module EHEN-C1, the reporter enzyme ec: 6.1.1.3, threonyl-tRNA synthetase (TARS), was an autoantigen in the autoimmune disorder myositis, and borrelidin, which was a potent inhibitor of TARS, inhibits angiogenesis. TARS thus provided a potential target for detecting or interdicting disease-related inflammatory or angiogenic responses [32]. And the reporter enzyme ec: 3.1.4.37 (2′,3′-cyclic-nucleotide 3′-phosphodiesterase), which was enriched on the function classification related lipid, regulates intracellular cAMP levels, which might represent novel therapeutic agents to limit angiogenesis in complex human diseases [33].

Because coronary heart disease is caused by abnormal lipid metabolism, so the GO terms associated with lipid metabolism can be direct evidence between reporter enzymes and CHD. We were more interested in related programmed cell death reporter enzymes and modules.

### 3.4. Literature Retrieval and Functional Annotation of the Module Related to Programmed Cell Death

**Figure 3:**
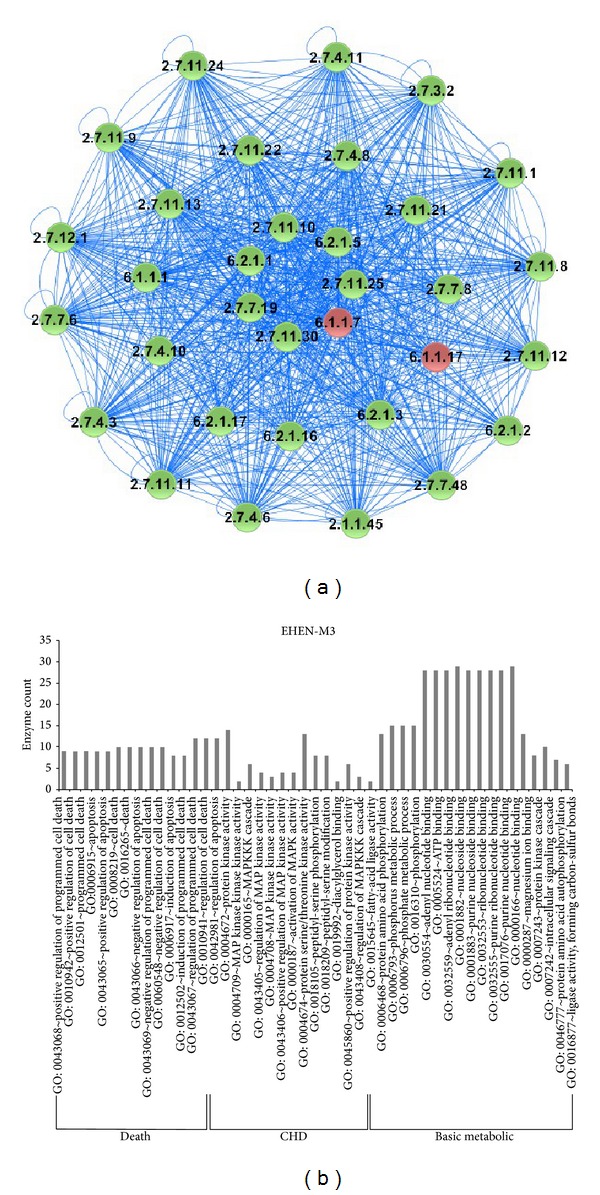
The module EHEN-M3 and the result of the functional enrichment. (a) The module 3 in EHEN-M. (b) The result of the functional enrichment.

The module 3 in mitochondria subnetwork (EHEN-M3) was a module which enriched to not only the basic metabolic function class, the related CHD pathogenesis, but also the related programmed cell death function class. It included 33 enzymes (2 reporter enzymes ec: 6.1.1.7 and ec: 6.1.1.17 and 31 others) (Figure 3(a)). Through GO enrichment analysis, the genes of these enzymes in the module enriched 47 GO terms, including 15 GO terms related programmed cell death, 14 GO terms related CHD, and 18 GO terms related basic metabolic (Figure 3(b)). In this module, we were interested in the part of programmed cell death. For the reporter enzyme ec: 6.1.1.17 (glutamate—tRNA synthetase), it was proved that glutamate—tRNA synthetase of* Bacillus subtilis* was known to result in the death of the host cell [34]. For the reporter enzyme ec: 6.1.1.7 (alanyl-tRNA synthetase), it was proved that a homozygous missense mutation in AARS2 causes perinatal or infantile cardiomyopathy with near-total combined mitochondrial respiratory chain deficiency in the heart, in which AARS2 is identified to encode mitochondrial alanyl-tRNA synthetase [35]. We found there were 13 enzymes enriched to GO terms related programmed cell death, and more of them belonged to serine/threonine kinases. For instance, the enzyme ec: 2.7.11.10, the inhibitor of NF-*κ*B kinase subunit *β* (IKK*β*), formed a transduction complex that controls the production of proinflammatory cytokines mediating cardiomyocyte hypertrophy, and activation of IKK*β* in turn enhances fetal gene expression and cardiomyocyte growth [36]. The enzyme ec: 2.7.11.22 (cyclin-dependent kinase) could interact with p27 in neonatal rat cardiomyocytes, which exerted antiapoptotic and growth-inhibitory effects, and may help to improve heart function and survival in rodents [37, 38].

## 4. Discussion

Traditionally, there are several ways to research the mechanism of CHD. Our approach focused on CHD in the programmed cell death perspective on the systems biology level. We constructed a correlative network of enzymes considering the cascade. The differences from the macrophages to the foam cell between disease patients and normal controls were calculated according to the expression level. We proposed a computational approach to select 7 modules based on reporter enzymes (2 in the EHEN, 2 in the EHEN-C, and 3 in the EHEN-M). Most of the modules could be enriched to the basic metabolic function class, the related CHD pathogenesis, and the related lipid function class.

The module EHEN-M3 was related to programmed cell death in the EHEN-M. Functional and structural integrity of mitochondria was essential for the physiological function of the cardiovascular system. Accumulation of mitochondrial DNA mutations has been linked to ischaemic heart disease, cardiomyopathy, and atherosclerotic vascular disease. Mitochondria are known to regulate apoptotic and autophagic pathways that have been shown to play an important role in the development of cardiomyopathy and atherosclerosis [39]. Our results also verified it from a new perspective of enzyme, which was a new research direction about CHD.

It was reported that macrophages and foam cells were associated with CHD in the aspect of programmed cell death. Wang et al. reported that macrophage with lipid growing eventually formed foam cells until death. A large pool of bubble formation and programmed cell death eventually developed into a typical plaque [40, 41]. More importantly, the reporter enzymes were obtained from macrophages and foam cells, which might be isolated from the peripheral blood of patient. Modules could be identified based on these reporter enzymes to reflect the disease state.

Deficiency or alterations in metabolic functions were known to be involved in CHD. Enzyme proteins and chemical compounds were connected in metabolic networks. The reconstruction of the enzyme network could illustrate cascade relationships of enzymes. The selected reporter enzymes and modules from each subnetwork were more closely associated with CHD. We hope that the research would be more comprehensive with more data accumulation, such as posttranslational regulatory data. Considering from this aspect, enzyme marker and modules could be searched and be used in disease detection in the future.

## Supplementary Material

Figure S1: Node distribution of the EHEN and sub networks. The horizontal axis and the vertical axis in each picture refer to node degree and numbers of genes.
Table S1: Topological characteristics of whole and sub networks of the EHEN.
Table S2: The list of the reporter enzymes of overall and sub network.
Table S3: The list of topological characteristics of reporter enzymes in each network. It is including average shortest, closeness centrality, degree, clustering, etc.
Table S4: The list is about modules based reporter enzymes in overall and part of sub-networks.

## Figures and Tables

**Table 1:** Module information of overall and part of subnetworks.

Network	Module count	RE count	Related function count			
			Basic metabolic	Lipid	CHD	Death
EHEN	2	4	32	18	9	—
EHEN-C	2	6	40	22	22	—
EHEN-M	3	5	51	13	35	15
