# Supplementary figures and images for: Identification of Modules Related to Programmed Cell Death in CHD Based on EHEN
Source: Biomed Res Int. 2014 Jul 15;2014:475379. doi: 10.1155/2014/475379 (PMC4123579; doi:10.1155/2014/475379)

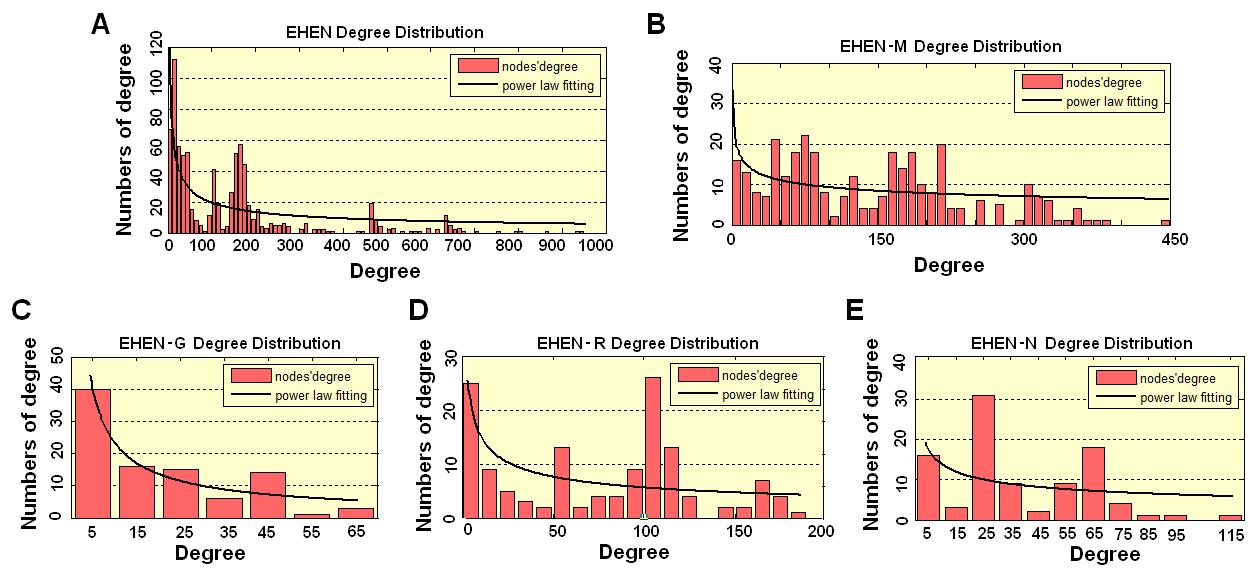

Supplement: Supplementary file 1 — Figure S1: Node distribution of the EHEN and sub networks. The horizontal axis and the vertical axis in each picture refer to node degree and numbers of genes. Table S1: Topological characteristics of whole and sub networks of the EHEN. Table S2: The list of the reporter enzymes of overall and sub network. Table S3: The list of topological characteristics of reporter enzymes in each network. It is including average shortest, closeness centrality, degree, clustering, etc. Table S4: The list is about modules based reporter enzymes in overall and part of sub-networks. [file 475379.f1.zip › Supplementary material Figure S1. Node distribution of the EHEN and sub networks.jpg]
