# Supplementary material for: Identification of Modules Related to Programmed Cell Death in CHD Based on EHEN
Source: Biomed Res Int. 2014 Jul 15;2014:475379. doi: 10.1155/2014/475379 (PMC4123579; doi:10.1155/2014/475379)
Supplement: Supplementary file 1 — Figure S1: Node distribution of the EHEN and sub networks. The horizontal axis and the vertical axis in each picture refer to node degree and numbers of genes. Table S1: Topological characteristics of whole and sub networks of the EHEN. Table S2: The list of the reporter enzymes of overall and sub network. Table S3: The list of topological characteristics of reporter enzymes in each network. It is including average shortest, closeness centrality, degree, clustering, etc. Table S4: The list is about modules based reporter enzymes in overall and part of sub-networks. [file 475379.f1.zip › Supplementary material Table S1. Topological characteristics of whole and sub networks of the EHEN .doc]

Table S1. Topological characteristics of whole and sub networks of the EHEN

| **Network** | **Number of nodes** | **Characteristic path length** | **Diameter** | **Avg.number of neighbors** | **Clustering coefficient** | **Number of edges** |
| --- | --- | --- | --- | --- | --- | --- |
| EHMN | 789 | 2.2219 | 5 | 70.464 | 0.34 | 55509 |
| EHMN-C | 688 | 2.134 | 5 | 136.892 | 0.553 | 48134 |
| EHMN-M | 320 | 1.92 | 5 | 89.575 | 0.593 | 22154 |
| EHMN-E | 79 | 2.157 | 7 | 29.797 | 0.583 | 1623 |
| EHMN-G | 95 | 2.643 | 7 | 12.884 | 0.452 | 855 |
| EHMN-N | 95 | 2.017 | 6 | 23.642 | 0.564 | 1687 |
| EHMN-R | 135 | 1.909 | 5 | 48.356 | 0.611 | 2 |
