# Supplementary material for: Identification of Modules Related to Programmed Cell Death in CHD Based on EHEN
Source: Biomed Res Int. 2014 Jul 15;2014:475379. doi: 10.1155/2014/475379 (PMC4123579; doi:10.1155/2014/475379)
Supplement: Supplementary file 1 — Figure S1: Node distribution of the EHEN and sub networks. The horizontal axis and the vertical axis in each picture refer to node degree and numbers of genes. Table S1: Topological characteristics of whole and sub networks of the EHEN. Table S2: The list of the reporter enzymes of overall and sub network. Table S3: The list of topological characteristics of reporter enzymes in each network. It is including average shortest, closeness centrality, degree, clustering, etc. Table S4: The list is about modules based reporter enzymes in overall and part of sub-networks. [file 475379.f1.zip › Supplementary material Table S2 The reporter enzymes of overall and sub network.doc]

Table S2. The reporter enzymes of overall and sub network.

| **EHEN** | **EHEN-C** | **EHEN-M** | **EHEN-E** | **EHEN-G** | **EHEN-N** | **EHEN-R** |
| --- | --- | --- | --- | --- | --- | --- |
| 4.4.1.20 | 6.1.1.7 | 6.1.1.7 | 3.1.4.37 | 2.4.1.102 | 2.7.1.127 | 2.4.1.119 |
| 6.1.1.15 | 2.4.1.119 | 6.3.5.4 | 2.4.1.90 | 2.7.8.11 | 3.2.1.10 | 2.7.8.15 |
| 3.2.1.48 | 2.8.2.29 | 1.14.13.13 | 3.1.1.34 | 2.4.1.135 | 2.7.1.74 |  |
| 2.7.1.127 | 3.1.4.37 | 1.14.15.6 | 2.4.1.38 | 3.1.4.45 | 2.4.2.31 |  |
| 6.3.5.4 | 6.1.1.18 | 2.1.4.1 | 2.4.2.31 | 2.4.1.38 |  |  |
| 5.3.2.1 | 2.7.11.14 | 2.7.4.9 |  |  |  |  |
| 2.1.3.2 | 1.14.15.6 | 6.1.1.15 |  |  |  |  |
|  | 3.2.1.10 | 2.7.1.30 |  |  |  |  |
|  | 3.1.4.45 | 6.1.1.3 |  |  |  |  |
|  | 1.13.11.6 | 6.1.1.17 |  |  |  |  |
|  | 6.3.4.5 | 1.13.11.34 | |  |  |  |
|  | 3.1.1.34 |  |  |  |  |  |
|  | 6.1.1.3 |  |  |  |  |  |
|  | 4.4.1.20 |  |  |  |  |  |
