# Supplementary material for: Identification of Modules Related to Programmed Cell Death in CHD Based on EHEN
Source: Biomed Res Int. 2014 Jul 15;2014:475379. doi: 10.1155/2014/475379 (PMC4123579; doi:10.1155/2014/475379)
Supplement: Supplementary file 1 — Figure S1: Node distribution of the EHEN and sub networks. The horizontal axis and the vertical axis in each picture refer to node degree and numbers of genes. Table S1: Topological characteristics of whole and sub networks of the EHEN. Table S2: The list of the reporter enzymes of overall and sub network. Table S3: The list of topological characteristics of reporter enzymes in each network. It is including average shortest, closeness centrality, degree, clustering, etc. Table S4: The list is about modules based reporter enzymes in overall and part of sub-networks. [file 475379.f1.zip › Supplementary material Table S3 Topological characteristics of reporter enzymes.doc]

Table S3. Topological characteristics of reporter enzymes in each network.

| **Network** | **Reporter**  **Enzyme** | **Average**  **Shortest**  **PathLength** | **Closeness**  **Centrality** | **Clustering**  **Coefficient** | **Degree** | **Neighborhood**  **Connectivity** | **Topological**  **Coefficient** |
| --- | --- | --- | --- | --- | --- | --- | --- |
| EHEN | 2.1.3.2 | 2.253807 | 0.443694 | 0.367647 | 17 | 164.2353 | 0.285627 |
|  | 5.3.2.1 | 2.969543 | 0.336752 | 0 | 5 | 38 | 0.336364 |
|  | 6.3.5.4 | 1.958122 | 0.510693 | 0.325429 | 104 | 226.7404 | 0.316235 |
|  | 3.2.1.48 | 2.02665 | 0.493425 | 0.365345 | 77 | 272.8831 | 0.39491 |
|  | 6.1.1.15 | 2.227157 | 0.449003 | 0.298246 | 19 | 163.2105 | 0.275229 |
|  | 2.7.1.127 | 2.313452 | 0.432255 | 0.577778 | 10 | 221.8 | 0.415356 |
|  | 4.4.1.20 | 2.598985 | 0.384766 | 0.5 | 5 | 133.6 | 0.398806 |
| EHEN-C | 6.1.1.18 | 2.045322 | 0.488921 | 0.652245 | 61 | 157.98 | 0.261556 |
|  | 3.1.4.37 | 1.513158 | 0.66087 | 0.659844 | 558 | 238.6071 | 0.350378 |
|  | 3.1.1.34 | 2.388889 | 0.418605 | 0.848485 | 13 | 277.3333 | 0.681409 |
|  | 4.4.1.20 | 1.710526 | 0.584615 | 0.815388 | 228 | 252.2279 | 0.378153 |
|  | 2.4.1.119 | 2.618421 | 0.38191 | 1 | 5 | 223 | 0.819853 |
|  | 2.7.11.14 | 2.146199 | 0.46594 | 0.967742 | 48 | 200.0968 | 0.361186 |
|  | 1.14.15.6 | 1.559942 | 0.64105 | 0.585246 | 406 | 229.6459 | 0.337715 |
|  | 6.3.4.5 | 2.040936 | 0.489971 | 0.556953 | 64 | 160.0185 | 0.265371 |
|  | 2.8.2.29 | 2.736842 | 0.365385 | 1 | 2 | 103.5 | 0.556452 |
|  | 6.1.1.3 | 2.054094 | 0.486833 | 0.679965 | 58 | 162.3125 | 0.270521 |
|  | 3.2.1.10 | 1.643275 | 0.608541 | 0.849003 | 390 | 255.7791 | 0.38176 |
|  | 6.1.1.7 | 1.817251 | 0.550282 | 0.353783 | 245 | 110.0245 | 0.170317 |
|  | 1.13.11.6 | 2.371345 | 0.421702 | 0.8 | 10 | 285.3 | 0.676066 |
|  | 3.1.4.45 | 1.647661 | 0.606921 | 0.852383 | 384 | 255.8872 | 0.383065 |
| EHEN-M | 6.1.1.3 | 2 | 0.5 | 0.816817 | 45 | 112.3514 | 0.399827 |
|  | 6.1.1.15 | 1.962264 | 0.509615 | 0.776671 | 46 | 112.1842 | 0.384193 |
|  | 6.3.5.4 | 1.352201 | 0.739535 | 0.423583 | 341 | 105.2201 | 0.334032 |
|  | 6.1.1.7 | 1.68239 | 0.594393 | 0.504927 | 180 | 92.85321 | 0.299526 |
|  | 2.7.4.9 | 2.003145 | 0.499215 | 0.866366 | 65 | 113.5946 | 0.405695 |
|  | 1.14.15.6 | 1.506289 | 0.663883 | 0.624204 | 223 | 125.8291 | 0.396937 |
|  | 1.14.13.13 | 1.59434 | 0.627219 | 0.637959 | 175 | 127.2406 | 0.405225 |
|  | 2.7.1.30 | 1.95283 | 0.512077 | 0.682788 | 60 | 111.7895 | 0.378947 |
|  | 6.1.1.17 | 1.896226 | 0.527363 | 0.5181 | 60 | 104.2885 | 0.348791 |
|  | 2.1.4.1 | 2.103774 | 0.475336 | 0.304094 | 28 | 65.31579 | 0.24701 |
|  | 1.13.11.34 | 2.103774 | 0.475336 | 0.809524 | 7 | 158.1429 | 0.568859 |
| EHEN-E | 3.1.1.34 | 2.118421 | 0.47205 | 1 | 7 | 49.66667 | 0.814208 |
|  | 2.4.2.31 | 2.197368 | 0.45509 | 1 | 9 | 31.75 | 0.557018 |
|  | 2.4.1.38 | 2.394737 | 0.417582 | 1 | 2 | 24.5 | 0.544444 |
|  | 2.4.1.90 | 2.118421 | 0.47205 | 1 | 2 | 48 | 0.738462 |
|  | 3.1.4.37 | 1.276316 | 0.783505 | 0.714478 | 88 | 40.49091 | 0.532775 |
| EHEN-G | 2.4.1.38 | 2.218391 | 0.450777 | 0.666667 | 3 | 28.33333 | 0.422886 |
|  | 2.7.8.11 | 2.172414 | 0.460317 | 0.564103 | 14 | 20.53846 | 0.336696 |
|  | 3.1.4.45 | 1.747126 | 0.572368 | 0.812308 | 41 | 26 | 0.313253 |
|  | 2.4.1.102 | 2.195402 | 0.455497 | 0.6 | 5 | 21.8 | 0.325373 |
|  | 2.4.1.135 | 2.264368 | 0.441624 | 0.666667 | 3 | 23 | 0.365079 |
| EHEN-N | 2.7.1.74 | 1.966667 | 0.508475 | 0.879121 | 24 | 34.28571 | 0.428571 |
|  | 2.7.1.127 | 2 | 0.5 | 0.871795 | 22 | 35.76923 | 0.45858 |
|  | 2.4.2.31 | 2.344444 | 0.42654 | 1 | 8 | 18.25 | 0.325893 |
|  | 3.2.1.10 | 1.633333 | 0.612245 | 0.908962 | 62 | 38.39474 | 0.451703 |
| EHEN-R | 2.4.1.119 | 2.458647 | 0.406728 | 1 | 5 | 61.5 | 0.866197 |
|  | 2.7.8.15 | 2.112782 | 0.47331 | 0.490909 | 17 | 34.72727 | 0.324554 |
