# Supplementary material for: Identification of Modules Related to Programmed Cell Death in CHD Based on EHEN
Source: Biomed Res Int. 2014 Jul 15;2014:475379. doi: 10.1155/2014/475379 (PMC4123579; doi:10.1155/2014/475379)
Supplement: Supplementary file 1 — Figure S1: Node distribution of the EHEN and sub networks. The horizontal axis and the vertical axis in each picture refer to node degree and numbers of genes. Table S1: Topological characteristics of whole and sub networks of the EHEN. Table S2: The list of the reporter enzymes of overall and sub network. Table S3: The list of topological characteristics of reporter enzymes in each network. It is including average shortest, closeness centrality, degree, clustering, etc. Table S4: The list is about modules based reporter enzymes in overall and part of sub-networks. [file 475379.f1.zip › Supplementary material Table S4. Modules based reporter enzymes in overall and part of sub-networks .doc]

Table S4. Modules based reporter enzymes in overall and part of sub-networks.

| Network | Network size | Module | Module size | Enzymes |
| --- | --- | --- | --- | --- |
| EHEN | 789 | EHEN 1 | 33 | ec：6.3.5.4*, ec：2.3.1.4, ec：6.3.5.5, ec：2.3.1.37, ec：2.4.2.19, ec：2.7.4.3, ec：2.1.1.45, ec：4.1.1.28, ec：2.4.2.8, ec：2.3.1.174, ec：6.1.1.17, ec：2.3.1.29, ec：6.2.1.17, ec：2.3.1.6, ec：6.3.2.12, ec：6.3.5.2, ec：3.6.1.6, ec：3.5.4.6, ec：2.3.1.42, ec：2.7.7.18, ec：2.3.1.57, ec：6.2.1.1, ec：2.7.1.147, ec：2.7.1.24, ec：2.7.4.10, ec：2.3.1.65, ec：2.3.1.7, ec：2.3.1.50, ec：6.3.5.3, ec：3.5.4.17, ec：6.3.2.17, ec：2.7.7.8, ec：2.7.7.1 |
|  |  | EHEN 2 | 227 | ec：2.1.3.2*, ec：3.2.1.48*, ec：6.1.1.15*, ec：2.7.3.2, ec：2.4.1.79, ec：4.1.2.13, ec：2.1.3.3, ec：3.2.1.20, ec：3.5.1.26, ec：3.2.1.23, ec：3.5.1.23, ec：2.1.2.1, ec：2.7.1.21, ec：1.3.99.12, ec：5.4.2.5, ec：2.7.1.60, ec：3.1.6.1, ec：6.1.1.16, ec：6.3.4.5, ec：1.14.11.2, ec：2.1.2.2, ec：3.6.1.8, ec：2.4.1.142, ec：3.2.1.31, ec：3.2.1.46, ec：2.7.1.67, ec：3.1.4.12, ec：3.1.2.6, ec：2.3.1.61, ec：1.4.3.3, ec：3.5.3.11, ec：6.1.1.11, ec：2.7.8.2, ec：2.7.1.82, ec：3.1.6.8, ec：4.1.2.27, ec：2.7.7.15, ec：6.1.1.12, ec：2.4.1.186, ec：2.4.1.198, ec：3.1.3.2, ec：2.7.1.59, ec：3.1.1.3, ec：4.4.1.13, ec：1.5.3.11, ec：1.13.11.31, ec：4.1.2.4, ec：1.3.99.10, ec：2.7.8.15, ec：3.1.4.11, ec：1.4.3.16, ec：5.3.1.1, ec：1.4.3.2, ec：2.5.1.16, ec：3.5.1.15, ec：4.1.1.9, ec：1.13.11.11, ec：3.5.99.6, ec：2.5.1.22, ec：4.3.1.19, ec：3.6.1.19, ec：3.1.4.45, ec：2.8.2.2, ec：3.1.3.9, ec：2.7.1.32, ec：2.8.2.11, ec：3.6.2.2, ec：2.4.1.11, ec：1.5.3.1, ec：2.4.1.90, ec：1.13.11.33, ec：1.6.5.3, ec：1.5.99.2, ec：3.4.11.4, ec：3.1.3.37, ec：2.4.1.80, ec：2.4.1.62, ec：2.6.1.16, ec：1.4.3.10, ec：2.1.1.5, ec：4.1.1.17, ec：1.4.4.2, ec：2.7.7.30, ec：2.7.9.3, ec：3.1.1.23, ec：5.4.2.2, ec：2.3.1.43, ec：2.7.1.107, ec：2.6.1.7, ec：3.5.1.89, ec：2.7.1.138, ec：1.3.99.7, ec：2.7.1.48, ec：2.4.1.117, ec：2.4.1.88, ec：1.14.11.8, ec：3.2.1.45, ec：3.2.1.51, ec：2.7.1.30, ec：2.7.4.8, ec：6.3.2.3, ec：2.6.1.40, ec：6.3.2.5, ec：1.3.3.6, ec：5.3.1.8, ec：3.1.4.50, ec：2.1.1.2, ec：4.1.1.45, ec：6.1.1.19, ec：4.1.1.29, ec：4.3.2.1, ec：1.1.99.1, ec：1.3.99.13, ec：3.2.1.10, ec：2.8.3.5, ec：3.2.1.22, ec：1.17.4.1, ec：1.3.99.3, ec：1.4.3.19, ec：3.5.1.25, ec：3.5.1.9, ec：3.1.3.4, ec：5.1.3.8, ec：6.4.1.4, ec：3.7.1.3, ec：5.1.3.2, ec：3.6.1.28, ec：2.5.1.46, ec：3.1.3.11, ec：1.1.99.5, ec：1.3.99.5, ec：2.7.4.9, ec：3.2.1.21, ec：5.1.3.3, ec：3.1.6.2, ec：4.1.3.3, ec：1.5.5.1, ec：6.1.1.10, ec：2.7.7.2, ec：3.1.1.5, ec：2.8.1.2, ec：6.4.1.3, ec：2.7.7.4, ec：3.1.4.43, ec：3.1.3.25, ec：2.7.1.6, ec：3.2.1.14, ec：2.7.1.52, ec：1.14.11.18, ec：2.7.7.12, ec：1.3.5.1, ec：3.1.4.4, ec：3.4.11.5, ec：2.3.2.4, ec：2.7.6.1, ec：5.3.1.9, ec：2.1.4.1, ec：3.1.3.46, ec：2.4.1.17, ec：5.3.1.6, ec：2.3.1.135, ec：3.2.1.33, ec：5.4.2.7, ec：1.14.99.1, ec：2.7.4.15, ec：1.13.11.6, ec：3.2.1.1, ec：2.2.1.2, ec：2.3.1.15, ec：2.7.1.91, ec：3.5.4.5, ec：2.7.1.3, ec：2.7.1.137, ec：2.7.1.105, ec：2.4.1.83, ec：3.6.1.18, ec：2.4.1.45, ec：2.7.8.11, ec：3.6.1.43, ec：2.7.7.41, ec：2.7.1.33, ec：3.5.3.1, ec：2.7.1.26, ec：2.1.1.17, ec：3.1.3.75, ec：2.4.1.109, ec：4.1.1.65, ec：1.14.11.1, ec：2.7.1.11, ec：2.4.1.141, ec：2.3.2.8, ec：2.7.7.9, ec：6.1.1.7, ec：2.7.1.108, ec：2.4.1.132, ec：4.1.1.36, ec：6.1.1.14, ec：3.1.1.4, ec：1.14.11.16, ec：2.1.1.20, ec：3.2.1.3, ec：3.6.1.13, ec：3.1.3.3, ec：6.3.2.6, ec：4.1.1.11, ec：2.7.1.40, ec：5.4.99.2, ec：3.5.1.28, ec：2.7.7.3, ec：3.2.1.108, ec：6.3.4.4, ec：2.1.1.13, ec：4.4.1.8, ec：4.4.1.16, ec：2.4.1.18, ec：3.2.2.19, ec：2.4.1.228, ec：3.5.1.14, ec：2.7.7.14, ec：3.6.1.23, ec：2.7.6.2, ec：2.2.1.6, ec：6.3.4.13, ec：3.5.4.12, ec：1.13.11.20, ec：4.4.1.5, ec：3.5.1.8, |
| EHEN-C | 688 | EHEN-C 1 | 79 | ec：6.1.1.3*, ec：6.1.1.18*, ec：6.3.4.5*, ec：6.1.1.6, ec：2.3.1.4, ec：2.6.1.13, ec：2.4.2.7, ec：4.6.1.1, ec：6.1.1.22, ec：2.4.2.19, ec：2.3.3.8, ec：6.1.1.5, ec：2.3.1.15, ec：6.1.1.15, ec：2.3.1.87, ec：6.3.4.10, ec：6.3.2.19, ec：2.7.7.41, ec：2.4.2.22, ec：2.7.7.6, ec：2.6.1.16, ec：6.1.1.16, ec：2.6.1.19, ec：2.6.1.22, ec：1.14.11.1, ec：2.5.1.29, ec：2.3.1.20, ec：2.5.1.8, ec：4.1.1.15, ec：2.4.2.10, ec：6.1.1.9, ec：2.6.1.2, ec：2.7.7.30, ec：2.7.7.52, ec：6.3.4.9, ec：2.7.1.24, ec：2.7.7.9, ec：2.3.1.51, ec：2.1.2.5, ec：2.3.1.61, ec：6.1.1.14, ec：2.3.1.97, ec：6.1.1.11, ec：2.5.1.10, ec：2.6.1.42, ec：2.3.1.48, ec：6.1.1.10, ec：2.7.7.2, ec：6.3.1.2, ec：2.3.1.5, ec：2.7.7.4, ec：2.5.1.1, ec：2.7.7.3, ec：2.7.7.15, ec：6.1.1.12, ec：6.1.1.17, ec：2.3.1.6, ec：6.3.2.12, ec：2.3.1.42, ec：2.3.1.26, ec：2.7.7.7, ec：2.3.1.21, ec：6.3.4.11, ec：2.7.7.14, ec：2.3.1.12, ec：6.3.4.15, ec：2.7.7.23, ec：2.6.1.1, ec：2.6.1.52, ec：2.3.1.7, ec：2.3.1.50, ec：6.1.1.19, ec：6.1.1.2, ec：6.1.1.20, ec：6.3.2.17, ec：6.1.1.4, ec：6.1.1.21, ec：2.7.7.48, ec：2.7.7.1, |
|  |  | EHEN-C 2 | 135 | ec：3.2.1.10*, ec：3.1.4.37*, ec：3.1.4.45*, ec：4.2.1.-, ec：3.2.1.114, ec：3.1.4.17, ec：1.1.1.205, ec：1.14.99.9, ec：1.11.1.12, ec：1.11.1.8, ec：3.2.1.22, ec：1.2.1.29, ec：1.14.14.1, ec：3.4.21.35, ec：1.3.1.-, ec：1.17.4.1, ec：3.2.1.23, ec：3.5.1.23, ec：1.14.15.4, ec：3.5.1.9, ec：3.1.3.4, ec：1.2.1.12, ec：3.1.1.13, ec：3.7.1.3, ec：3.5.2.7, ec：3.5.4.16, ec：3.1.1.32, ec：1.1.1.1, ec：3.2.1.31, ec：4.2.1.49, ec：1.2.1.16, ec：1.2.3.1, ec：3.1.6.2, ec：1.1.1.35, ec：1.13.11.34, ec：3.1.1.5, ec：3.1.4.43, ec：1.2.1.3, ec：3.1.2.2, ec：1.2.1.24, ec：2.5.1.6, ec：4.2.1.46, ec：3.2.1.18, ec：3.4.15.1, ec：3.1.1.3, ec：1.14.-.-, ec：3.2.1.14, ec：3.2.1.106, ec：6.3.5.1, ec：3.1.3.-, ec：1.13.11.31, ec：3.4.19.6, ec：4.2.1.2, ec：3.1.4.11, ec：3.4.17.1, ec：4.2.1.3, ec：1.16.3.1, ec：1.4.3.4, ec：3.6.1.22, ec：3.1.4.4, ec：3.5.1.15, ec：1.5.1.9, ec：3.4.11.5, ec：3.6.1.9, ec：3.1.3.16, ec：3.1.3.9, ec：1.2.1.39, ec：3.6.1.-, ec：3.2.1.33, ec：3.1.-, ec：3.1.3.48, ec：1.14.99.1, ec：2.4.1.-, ec：3.2.1.52, ec：3.2.1.1, ec：3.4.14.9, ec：3.4.21.39, ec：3.1.1.1, ec：4.2.1.11, ec：3.5.4.10, ec：1.5.99.2, ec：3.3.2.10, ec：3.6.1.5, ec：1.11.1.9, ec：3.1.3.75, ec：2.3.1.85, ec：3.1.1.2, ec：3.3.2.6, ec：4.2.1.61, ec：1.5.1.6, ec：3.5.1.-, ec：3.1.3.67, ec：4.3.1.17, ec：3.2.2.5, ec：4.2.1.75, ec：1.2.1.36, ec：3.6.1.45, ec：3.4.13.19, ec：3.4.21.26, ec：3.5.2.2, ec：3.1.-.-, ec：3.1.1.4, ec：3.1.2.15, ec：2.6.1.7, ec：3.2.1.3, ec：3.5.1.89, ec：3.1.1.29, ec：3.5.1.4, ec：3.4.24.15, ec：3.2.1.84, ec：3.5.2.3, ec：4.2.1.10, ec：2.5.1.18, ec：1.8.4.11, ec：1.1.1.22, ec：1.14.17.1, ec：2.3.1.154, ec：1.4.3.6, ec：1.2.1.19, ec：1.2.1.4, ec：3.4.24.16, ec：3.2.2.19, ec：3.4.15.-, ec：3.5.1.14, ec：1.11.1.7, ec：1.1.1.170, ec：3.1.3.66, ec：2.3.2.2, ec：4.2.1.96, ec：3.1.1.31, ec：3.2.1.113, ec：3.1.2.22, ec：1.5.1.12, ec：1.2.1.5, ec：3.5.4.9, |
| EHEN-M | 320 | EHEN-M 1 | 33 | ec：6.3.5.4*, ec：1.14.13.13*, ec：2.3.3.10, ec：3.1.2.4, ec：1.3.3.3, ec：1.3.3.4, ec：3.4.24.16, ec：3.5.1.23, ec：1.5.99.2, ec：1.4.3.4, ec：1.17.4.1, ec：4.2.1.55, ec：2.5.1.18, ec：3.1.3.66, ec：1.14.19.-, ec：1.14.17.1, ec：1.14.15.5, ec：1.13.11.31, ec：3.1.3.16, ec：1.11.1.12, ec：4.2.1.3, ec：3.1.3.48, ec：4.2.1.-, ec：3.5.1.63, ec：3.1.3.-, ec：4.2.1.74, ec：4.2.1.18, ec：1.11.1.9, ec：4.2.1.2, ec：3.1.1.29, ec：2.3.3.1, ec：1.1.99.1, ec：2.3.1.41, |
|  |  | EHEN-M 2 | 38 | ec：1.14.15.4, ec：3.1.1.4, ec：1.1.1.205, ec：1.14.99.9, ec：1.5.99.8, ec：1.2.1.31, ec：1.14.13.30, ec：1.5.1.9, ec：6.4.1.4, ec：1.2.1.47, ec：1.14.-.-, ec：1.14.99.10, ec：2.6.1.7, ec：1.2.1.3, ec：1.5.1.7, ec：1.8.1.4, ec：1.16.3.1, ec：1.14.13.-, ec：1.5.1.8, ec：1.14.13.9, ec：1.14.15.6*, ec：1.11.1.7, ec：2.3.1.154, ec：4.2.1.17, ec：1.5.1.12, ec：1.2.1.19, ec：1.14.13.15, ec：1.2.4.4, ec：3.5.4.9, ec：1.2.1.16, ec：3.1.2.2, ec：2.1.2.10, ec：1.2.1.5, ec：1.14.99.-, ec：1.3.99.2, ec：1.2.1.24, ec：1.4.1.3, ec：6.3.4.3, |
|  |  | EHEN-M 3 | 33 | ec：6.1.1.7*, ec：6.1.1.17*, ec：6.2.1.16, ec：6.2.1.1, ec：2.7.11.8, ec：2.7.7.19, ec：2.7.7.48, ec：6.2.1.17, ec：2.7.7.8, ec：2.7.3.2, ec：6.2.1.2, ec：2.7.4.10, ec：2.7.4.6, ec：6.2.1.3, ec：6.2.1.5, ec：2.1.1.45, ec：6.1.1.1, ec：2.7.11.1, ec：2.7.4.3, ec：2.7.4.8, ec：2.7.11.13, ec：2.7.4.11, ec：2.7.11.25, ec：2.7.11.12, ec：2.7.11.30, ec：2.7.11.22, ec：2.7.7.6, ec：2.7.11.11, ec：2.7.11.9, ec：2.7.11.24, ec：2.7.11.21, ec：2.7.12.1, ec：2.7.11.10, |

* were reporter enzymes.
